# Impact of major different variants of papillary thyroid microcarcinoma on the clinicopathological characteristics: the study of 1041 cases

**DOI:** 10.1007/s10147-017-1170-6

**Published:** 2017-07-25

**Authors:** Jingtai Zhi, Jingzhu Zhao, Ming Gao, Yi Pan, Jianghua Wu, Yigong Li, Dapeng Li, Yang Yu, Xiangqian Zheng

**Affiliations:** 1 Department of Thyroid and Neck Tumor, National Clinical Research Center for Cancer, Key Laboratory of Cancer Prevention and Therapy, Tianjin’s Clinical Research Center for Cancer, Tianjin Medical University Cancer Institute and Hospital, Tianjin, 300060 People’s Republic of China; 2 Department of Pathology, National Clinical Research Center for Cancer, Key Laboratory of Cancer Prevention and Therapy, Tianjin’s Clinical Research Center for Cancer, Tianjin Medical University Cancer Institute and Hospital, Tianjin, 300060 People’s Republic of China

**Keywords:** Papillary thyroid microcarcinoma, Variant, Clinicopathological characteristics

## Abstract

**Background:**

The incidence of papillary thyroid microcarcinoma (PTMC) has been increasing globally in the past few decades. PTMC does not have a distinctive morphology that results in differences in biological behavior. The aim of this study was to classify PTMCs according to the morphological features and explore the relationship with clinicopathological characteristics. Additionally, we sought to evaluate whether different variants of PTMC can be an independent predictor for lymph mode metastasis when considering other risk factors.

**Methods:**

Between December 2014 and December 2015, 1041 PTMC cases undergoing surgical resection at Tianjin Medical University Cancer Institute and Hospital were reviewed retrospectively. Statistical analysis was performed to investigate the independent factors for lymph node metastasis in PTMC.

**Results:**

Conventional variant PTMC (CPTMC), follicular variant PTMC (FPTMC), and encapsulated variant PTMC (EnPTMC) were major variants in PTMC, collectively accounting for 96.7% of the entire PTMC cohort.There were significant differences in clinicopathological characteristics among the three major variants. The frequency of aggressive parameters was significantly different among the three variants, including tumor size, minimal extrathyroidal extension (minimal ETE), and lymph node metastasis (all *P* < 0.05), being highest in CPTMC, lowest in EnPTMC, and intermediate in FPTMC. FPTMC (OR = 0.642, *P* = 0.003) and EnPTMC (OR = 0.540, *P* = 0.041) were independent protective factors for lymph node metastasis (LNM). In contrast, male gender (OR = 1.836, *P* = 0.000), age less than 45 years (OR = 1.457, *P* = 0.009), tumor size greater than 0.5 cm (OR = 1.453, *P* = 0.007), calcification (OR = 1.465, *P* = 0.016), minimal ETE (OR = 1.801, *P* = 0.001), and multifocality (OR = 1.721, *P* = 0.000) were independent risk factors for LNM.

**Conclusions:**

The present study demonstrates the distinct biological behaviors of the three major PTMC variants and establishes an aggressive order of CPTMC ≫ FPTMC > EnPTMC. It is necessary to take into consideration variant-related risks and other independent predictors for the determination of lymphadenectomy in patients with PTMC.

## Introduction

Papillary thyroid microcarcinoma (PTMC), defined as papillary thyroid carcinoma (PTC) 10 mm or less in diameter, has been dramatically rising in incidence during the past few decades, accounting for nearly half the increase in PTC [1–4]. PTMC is generally regarded as an indolent disease but does pose a risk for local recurrence and distant metastasis [5, 6]. Identification of aggressive cases from favorable ones is necessary for the patient-tailored treatment of PTMC.

PTMC does not have a distinctive morphology, which means that all cellular features and growth patterns that can be found in other variants of PTC can be observed in PTMC [7, 8]. In PTC, the variant-related differences in biological behavior and prognosis are very large. Compared with the conventional variant, solid variant, diffuse sclerosing variant, and tall cell variant are recommended as the aggressive types, and follicular variant and the Warthin-like variant are considered favorable [9–14], which has further led to variant-related changes in PTC treatment [11, 13, 15, 16]. Considering the findings of these studies, the appearance of other variants in PTMC may incur variant-relevant risk, which has a profound impact on the treatment strategies. The purpose of this study was to classify the PTMCs according to the nature of the tumor boundaries, distinct architecture, or cellular characteristics and explore the clinicopathological characteristics of different variants of PTMC. Additionally, we sought to evaluate whether different variants of PTMC can be an independent predictor for lymph mode metastasis when considering other risk factors.

## Materials and methods

This retrospective cohort review research was supported by the academic ethics board, and informed consent was obtained from all patients to allow their information to be used for the study.

### Patients

We consecutively collected 1041 patients from all the PTMC cases between December 2014 and December 2015 in our hospital. Exclusion criteria were (1) patients had other malignancy, (2) no lymph node was resected, (3) distant metastasis, (4) patients with a history of previous operation for PTC, (5) largest tumor greater than 1 cm, and (6) age less than 18 years. All cases were grouped according to the subtype of the largest tumor. These chosen patients were mostly asymptomatic, and their lesions were accidently discovered during the annual physical examination. Once the nodules were suspected for malignancy by fine-needle aspiration biopsy (FNAB) or ultrasound (US), either thyroid lobectomy or total/near total thyroidectomy combined with prophylactic central neck dissection was performed in all patients. Patients who had preoperatively or intraoperatively proven lateral neck metastasis by FNAB or biopsy underwent therapeutic lateral neck dissection.

### Pathological examination

Both intraoperative frozen and postoperative paraffin sections were submitted to two experienced endocrine pathologists (Yi Pan and Jianghua Wu) to establish the diagnosis of PTMC and other characteristic pathological parameters, including histopathological types, multifocality, coexistence of Hashimoto’s thyroiditis (HT), minimal extrathyroidal extension (minimal ETE), status of lymph node metastasis (LNM), and tumor size, fibrosis, and calcification of the largest tumor. All the pathological parameters were identified according to Rosai and Ackerman’s surgical pathology, 10th edition. Representative sections were examined in a blinded fashion, and the concordance rate was 97%. The final diagnoses of discordant or rare-variant cases were based on the consensus of two pathologists.

### Statistical analysis

Differences among variants were compared by the chi-square test. Univariate and multivariate logistic regression models were used to evaluate independent predictors for lymph mode metastasis in PTMC. *P* < 0.05 was assumed as statistical significance. SPSS version 22.0.0 (IBM, Armonk, NY, USA) was used to perform the statistical analysis.

## Results

**Fig. 1:**
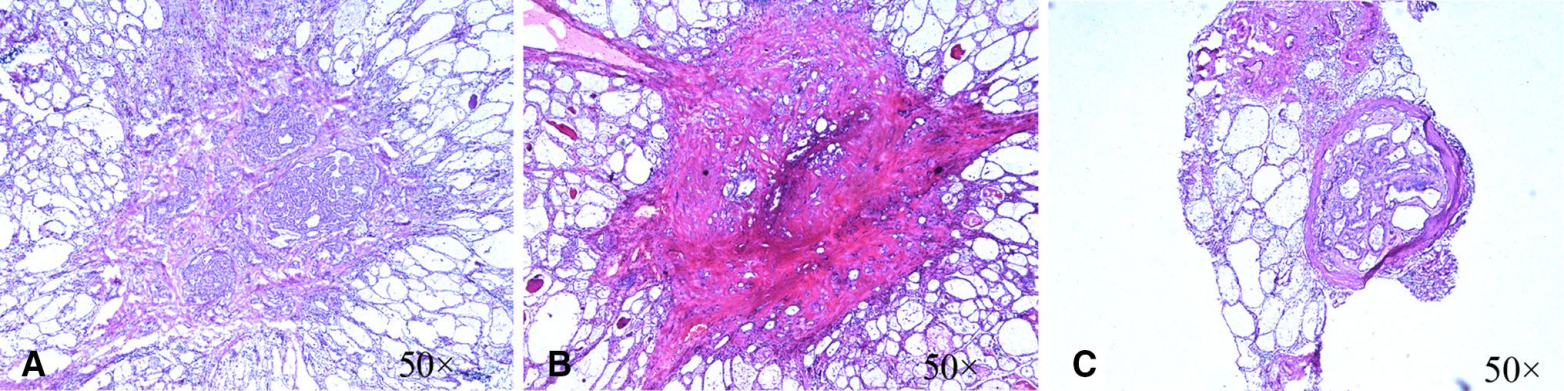
**a** Conventional variant: characterized by papillary architecture, *Φ* = 2 mm. **b** Follicular variant: a papillary carcinoma composed entirely or almost entirely of follicles, *Φ* = 3 mm. **c** Encapsulated variant: a papillary carcinoma totally surrounded by a capsule, *Φ* = 1 mm

There were 1041 PTMC patients enrolled in this study. The major variants of PTMC, including conventional variant (CPTMC, 471 cases, 45.2%; Fig. 1a), follicular variant (FPTMC, 454 cases, 43.6%; Fig. 1b), and encapsulated variant (EnPTMC, 82 cases, 7.9%; Fig. 1c), collectively accounted for 96.7% of the entire PTMC cohort. Other variants of PTMC (e.g., Warthin-like variant, solid variant, oncocytic variant, tall cell variant, or trabecular variant) were rare (collectively, 34 cases, 3.3%), so we only analyzed the clinicopathological characteristics of the 1007 cases with the major variants. The sex ratio was 1:3.86 (male:female, 207:800). The age range was 18–73 (mean, 46 ± 9.5) years. The size of the largest tumor was 5.7 ± 1.8 (mean ± SD) mm. The rate of fibrosis and calcification was 98.5% and 31.7%, respectively. Multifocal tumors were found in 364 cases (Table 1). Minimal ETE was seen in 200 (19.9%) patients, including 59 in vascular, 120 in fat, 8 in striated muscle, 1 in nerve, and 12 multiple minimal ETE. HT was present in 26.3% of the patients. The frequency of LNM was 26.3%; among these, 275 were N1a and 40 were N1b (Table 2). Most patients (94.6%) underwent prophylactic central neck dissection; in only 54 patients was central + lateral neck dissection performed.

**Table 1:** Distribution of variants among 1041 patients between December 2014 and December 2015

Subtype	*N*	Present
Conventional	471	45.2
Follicular	454	43.6
Encapsulated	82	7.9
Warthin-like	17	1.6
Solid	3	0.3
Oncocytic	3	0.3
Tall cell	6	0.6
Trabecular	5	0.5

**Table 2:** Clinicopathological characteristics of patients with three major variants of papillary thyroid microcarcinoma (PTMC)

Characteristics	*N* (mean ± SD)	Present
Gender		
Female	800	79.4
Male	207	20.6
Age (years)	46 ± 9.5	
≥45	558	55.4
<45	449	44.6
Tumor size	0.57 ± 0.18	
>5 mm	510	50.6
≤5 mm	497	49.4
Fibrosis		
Absent	15	1.5
Present	992	98.5
Calcification		
Absent	688	68.3
Present	319	31.7
Minimal ETE		
Absent	807	80.1
Present	200	19.9
Vascular	59	5.9
Fat	120	11.9
Striated muscle	8	0.8
Nerve	1	0.1
Multiple MEE	12	1.2
Coexistence of HT		
Absent	742	73.7
Present	265	26.3
Multifocality		
Absent	643	63.9
Present	364	36.1
Lymph node metastases		
Absent	692	68.7
Present	315	31.3
N-stage		
N0	692	68.7
N1a	275	27.3
N1b	40	4.0
Neck excision extent		
Central	953	94.6
Central + lateral	54	5.4

*Minimal ETE* minimal extrathyroidal extension, *HT* Hashimoto’s thyroiditis, *LNM* lymph node metastases

### Comparison of the clinicopathological data among three variants of PTMC

Comparisons of the clinicopathological features of the three PTMC variants are summarized in Table 3. Age, calcification, MEE, tumor size, and LNM showed significant differences among three variant-specific groups. The aggressive parameters, including MEE, tumor size, and lymph node metastasis, were highest in CPTMC, lowest in EnPTMC, and generally intermediate in FPTMC. In contrast with this order, the prevalence of age of 45 years or more and calcification was highest in EnPTMC, lowest in CPTMC, and generally intermediate in FPTMC. These patterns were further confirmed on pair-wise comparison (Table 4). Specifically, the prevalence of patient age of 45 years or more was similar between FPTMC and EnPTMC and significantly less common in CPTMC. The rate of calcification was similar between CPTMC and FPTMC and significantly higher in EnPTMC. Tumor size was similar between FPTMC and EnPTMC, and both were smaller than CPTMC. The incidence of minimal ETE showed no difference between CPTMC and FPTMC, but minimal ETE was significantly less commonly seen in EnPTMC than in CPTMC and FPTMC. The occurrence of LNM was significantly higher in CPTMC than in FPTMC and EnPTMC.

**Table 3:** Comparison of the clinicopathological data among three variants of PTMC

Characteristic	CPTMC	FPTMC	EnPTMC	*χ* ^2^	*P*
Gender					
Female	360 (76.4%)	373 (82.2%)	67 (81.7%)	4.920	0.085
Male	111 (23.6%)	81 (17.8%)	15 (18.3%)		
Age (years)					
≥45	239 (50.7%)	263 (57.9%)	56 (68.3%)	10.827	0.004
<45	232 (49.3%)	191 (42.1%)	26 (31.7%)		
Fibrosis					
Absent	11 (2.3%)	4 (0.9%)	0 (0.0%)	4.682	0.096
Present	460 (97.7%)	450 (99.1%)	82 (100%)		
Calcification					
Absent	351 (74.5%)	308 (67.8%)	29 (35.4%)	49.564	<0.001
Present	120 (25.5%)	146 (32.2%)	53 (64.6%)		
Minimal ETE					
Absent	370 (78.6%)	355 (78.2%)	82 (100%)	22.143	<0.001
Present	101 (21.4%)	99 (21.8%)	0 (0.0%)		
Coexistence of HT					
Absent	339 (72.0%)	344 (75.8%)	59 (72.0%)	1.857	0.395
Present	132 (28.0%)	110 (24.2%)	23 (28.0%)		
Multifocality					
Absent	302 (64.1%)	285 (62.8%)	56 (68.3%)	0.943	0.624
Present	169 (35.9%)	169 (37.2%)	26 (31.7%)		
Tumor size					
>5 mm	272 (57.7%)	204 (44.9%)	34 (41.5%)	18.200	<0.001
≤5 mm	199 (42.3%)	250(55.1%)	48 (58.5%)		
LNM					
Absent	296 (62.8%)	332 (73.1%)	64 (78.0%)	14.986	0.001
Present	175 (37.2%)	122 (26.9%)	18 (22.0%)		

*CPTMC* conventional variant PTMC, *FPTMC* follicular variant PTMC, *EnPTMC* encapsulated variant PTMC, *minimal ETE* minimal extrathyroidal extension, *HT* Hashimoto’s thyroiditis, *LNM* lymph node metastases

**Table 4:** Pairwise comparison of clinicopathological features among three variants of PTMC

Characteristic	CPTMC versus FPTMC	FPTMC versus EnPTMC	EnPTMC versus CPTMC
Age	0.028	0.078	0.003
Calcification	0.025	<0.001	<0.001
Minimal ETE	0.894	<0.001	<0.001
Tumor size	<0.001	0.560	0.006
LNM	0.001	0.351	0.008

*CPTMC* conventional variant PTMC, *FPTMC* follicular variant PTMC, *EnPTMC* encapsulated variant PTMC, *minimal ETE* minimal extra-thyroidal extension, *HT* Hashimoto’s thyroiditis, *LNM* lymph node metastases

### Correlation between clinicopathological features and LNM

Univariate logistic regression analysis indicated that gender, age, subtypes, MEE, multifocality, and tumor size were all strongly associated with the LNM in PTMC (*P* < 0.05). There was a significant difference in the occurrence of LNM between CPTMC with calcification and without calcification. In the other two subtypes, however, calcification was not remarkably correlated with incidence of LNM. Multiple logistic regression analysis indicated that male gender (OR, 1.896; 95% CI, 1.355–2.651), age45 years or less (OR, 1.393; 95% CI, 1.053–1.842), tumor size (OR, 1.453; 95% CI, 1.068–1.977), minimal ETE (OR, 1.801; 95% CI, 1.257–2.579), and multifocality (OR, 1.530; 95% CI, 1.129–2.074) were all significantly connected with the LNM in PTMC (*P* < 0.05). Compared with CPTMC, FPTMC showed significant differences (*P* = 0.006), which means that FPTMC (OR, 0.660; 95% CI, 0.492–0.885) was an independent protective factor for LNM. Although the *P* value >0.05, the OR of EnPTMC was 0.625, also indicating the indolent nature of EnPTMC (Table 5).

**Table 5:** Univariate analysis of lymph node metastasis and clinicopathological features in PTMC

Characteristic	LNM−	LNM+	*χ* ^2^	*P*
Gender				
Female	573	227	15.289	<0.001
Male	119	88		
Age (years)				
≥45	406	152	9.506	0.002
<45	286	163		
Subtypes				
CPTMC	296	175	14.986	0.001
FPTMC	332	122		
EnPTMC	64	18		
Fibrosis				
Absent	9	6	0.538	0.463
Present	683	309		
Calcification				
CPTMC				
Absent	232	119	6.239	0.012
Present	64	56		
FPTMC				
Absent	232	76	2.352	0.125
Present	100	46		
EnPTMC				
Absent	24	5	0.581	0.446
Present	40	13		
Minimal ETE				
Absent	585	222	26.890	0.000
Present	107	93		
Coexistence of HT				
Absent	505	237	0.571	0.450
Present	187	78		
Multifocality				
Absent	474	169	20.671	0.000
Present	218	146		
Tumor size				
>5 mm	311	199	28.788	0.000
≤5 mm	381	116		

*CPTMC* conventional variant PTMC, *FPTMC* follicular variant PTMC, *EnPTMC* encapsulated variant PTMC, *minimal ETE* minimal extrathyroidal extension, *HT* Hashimoto’s thyroiditis, *LNM* lymph node metastases

## Discussion

The American Thyroid Association (ATA) guidelines recommend thyroidectomy without prophylactic central neck dissection is appropriate for small (T1 or T2), noninvasive, clinically node-negative (cN0) PTC [15]. In cases whose largest tumor is >10 mm, this practice successfully keeps the balance of the risk caused by the enlarged surgery extent and the benefit of reduction in recurrence. In PTMC, however, the balance is remarkably disturbed by variant-specific differences without sufficient attention. Although PTMC is a subset of PTC, variants of PTMC differ from those of PTC in two respects: clinicopathological features and variant distribution, resulting in an unbalanced extent of surgery in PTMC patients (Table 6).

**Table 6:** Multivariate analysis of lymph node metastasis and clinicopathological features in PTMC

Characteristics	*B*	SE	Wald	*P*	OR	95% CI	
						Lower	Upper
Gender	0.640	0.171	13.964	0.000	1.896	1.355	2.651
Age	−0.331	0.143	5.381	0.020	1.393	1.053	1.842
Subtypes							
CPTMC			8.681	0.013	Reference		
FPTMC	−0.415	0.150	7.687	0.006	0.660	0.492	0.885
EnPTMC	−0.469	0.296	2.522	0.112	0.625	0.350	1.116
Tumor size	0.426	0.155	7.527	0.06	1.530	1.129	2.074
Fibrosis	−0.327	0.549	0.356	0.551	0.721	0.246	2.112
Minimal ETE	0.581	0.182	10.153	0.001	1.788	1.251	2.557
Coexistence of HT	−0.060	0.166	0.130	0.719	0.942	0.680	1.305
Multifocality	0.537	0.147	13.282	0.000	1.712	1.282	2.285
Constant	−1.400	0.644	4.725	0.030	0.247		

*CPTMC* conventional variant PTMC, *FPTMC* follicular variant PTMC, *EnPTMC* encapsulated variant PTMC, *minimal ETE* minimal extrathyroidal extension, *HT* Hashimoto’s thyroiditis

Encapsulated variant is defined as a papillary carcinoma totally surrounded by a capsule. In the present study, EnPTMC showed excellent biological behavior, being the lowest in aggressive parameters (tumor size and incidence of minimal ETE and LNM) among three variants. Because several authors have reported that age less than 45 years had a higher incidence of lymph node metastasis [17, 18], the age distribution also indicated the indolent nature of EnPTMC. The previous studies of PTC grouped the encapsulated variant according to the variant inside, underlining the benign behavior of the encapsulated follicular variant of PTC (E-FPTC) [19, 20]. One study reported that E-FPTC resembled the adenoma/follicular carcinoma group of tumors in its capsular/vascular invasive pattern and its propensity for lymph node metastasis whereas E-CPTC behaved more like conventional PTC [21]. In 2016, E-FPTC was categorized as low risk by the ATA [15]. In the present study, however, there was no significant difference in any respect between the encapsulated conventional variant of PTMC (E-CPTMC) and the encapsulated follicular variant of PTMC (not shown in the table), and both were shown as indolent. Overtreatment may occur in patients with EnPTMC (especially E-CPTMC), which needs further studies to determine the optimal extent of surgery.

Follicular variant is defined as a papillary carcinoma composed entirely or almost entirely of follicles. FPTMC was entirely intermediate in all parameters between CPTMC and EnPTMC. Except for the incidence of minimal ETE, all the aggressive parameters including age, tumor size distribution, and the occurrence rate of LNM in FPTMC was analogous to that of EnPTMC, remarkably differentiated from that of CPTMC. These findings are supported by the previous studies on PTC [9, 22]. In the present study, FPTMC accounted for 43.6% of the 1041 PTMCs, whose prevalence was nearly equal to that of CPTMC. This finding was particularly worth noting because several reports mentioned that the incidence of CPTC was 2 to 4.5 times higher than that of FPTC [9, 23, 24]. One reasonable hypothesis for the decrease in proportion of follicular variant from PTMC to PTC is that, compared with CPTMCs, fewer FPTMCs enlarge in tumor size and develop into FPTC. In spite of the absence of relevant evidence, this hypothesis may have some association with the phenomenon that quite a number of PTMCs enlarged slowly or almost did not enlarge [25, 26]. In our study, the size of tumor was significantly smaller in FPTMCs than in CPTMCs (44.9% vs. 55.7%, *P* < 0.001). The change in variant distribution indicates the specific growth pattern on FPTMC, deserving further studies and cautious selection in surgery extent.

In our univariate and multivariate analysis, LNM was significantly associated with male gender, age less than 45 years, tumor size, minimal ETE, and multifocality, which was supported by the recent study [6, 27, 28]. Compared with CPTMC, FPTMC was an independent protective factor for LNM, again demonstrating the indolent nature of this variant.

Calcification has been reported as a significant indicator for nonprogressive disease in PTMC [29], which was in contrast to our findings. In CPTMC, calcification was positively correlated with LNM, whereas in FPTMC and EnPTMC, no significant differences in LNM were found between tumors with or without calcification. Calcification in both subtypes was correlated with the unknown growth pattern but not with the occurrence of LNM. A cautious extent of surgery should be selected whether or not calcification is observed.

To investigate whether subtypes can be confirmed intraoperatively, differences in variant identification were compared between intraoperative frozen sections and postoperative paraffin sections. In intraoperative frozen sections, all the variants, except for the oncocytic variant, can be identified correctly according to the distinctive morphological characters, meaning that for almost all PTMCs, variants can be confirmed intraoperatively and thus used for the decision as to surgery extent.

There are three limitations in the present study. To clarify whether cases had LNM, we excluded the cases with no lymph node resected, which led to a slight rise in the rate of LNM. This issue was minimized by the rare occurrence of this situation. Also, the lower rate of LNM indicated that use of lymphadenectomy should be cautious in PTMC. The effect of surgery extent on data was another issue, being minimized by preoperative US of every patient. Because of color aberration, the oncocytic variant may be confused with other variants in frozen sections. As this variant does not have any prognostic implications in PTC [8] and was rare in PTMC, this limitation may not be important.

## Conclusion

The present study establishes the three major variants in PTMC and their aggressive order: CPTMC ≫ FPTMC > EnPTMC. For reasons of distinct clinicopathological features and variant distribution, variant identification has important clinical significance in determination of lymphadenectomy, having an effect on individual treatments in PTMC. Patients with variant-relevant risk and other predictors, including male gender, age less than 45 years, tumor size, minimal ETE, calcification, and multifocality, are recommended to undergo prophylactic lymph node dissection.

## Abbreviations

PTMC: Papillary thyroid microcarcinoma
PTC: Papillary thyroid carcinoma
FNAB: Fine-needle aspiration biopsy
LNM: Lymph node metastasis
Minimal ETE: Minimal extrathyroidal extension
HT: Hashimoto’s thyroiditis
CPTMC: Conventional variant PTMC
FPTMC: Follicular variant PTMC
EnPTMC: Encapsulated variant PTMC
E-FPTC: Encapsulated follicular variant of PTC
E-CPTC: Encapsulated conventional variant of PTC
